# Development of squamous cell carcinoma at the bite sites several years following suspected cobra (*Naja naja*) envenomings

**DOI:** 10.1080/15563650.2024.2432407

**Published:** 2024-12-03

**Authors:** Subramanian Senthilkumaran, José R. Almeida, Jarred Williams, Harry F. Williams, Ponniah Thirumalaikolundusubramanian, Sakthivel Vaiyapuri

**Affiliations:** a Manian Medical Centre, Erode, Tamil Nadu, India; b School of Pharmacy, University of Reading, Reading, UK; c Toxiven Biotech Private Limited, Coimbatore, Tamil Nadu, India; d The Tamil Nadu Dr M.G.R Medical University, Chennai, Tamil Nadu, India

**Keywords:** Cancer, cobra, long-term complications, *Naja naja*, snakebite envenoming, venom

## Abstract

**Introduction:**

Snakebite envenoming is a multidimensional issue that causes severe functional and life-challenging consequences among rural communities in tropical countries. Current research and treatments are largely focused on the acute effects of envenomation and short-term health outcomes. The knowledge of snakebite-induced long-term consequences is highly limited.

**Case series:**

We report the development of squamous cell carcinoma at the bite site several years later in four patients who are suspected to have been bitten by cobras (*Naja naja*). Following bites, the victims presented typical symptoms of cobra envenomings including ptosis, altered sensorium, and breathing difficulties. However, difficult-to-heal wounds were a chronic health sequelae with frequent desquamation cycles which led to squamous cell carcinoma. Surgery as the primary therapeutic approach was used for all patients to address this issue.

**Discussion:**

These patients highlight the importance of squamous cell carcinoma in previously damaged tissue from snakebites as a possible long-term consequence. This emphasises the need for surveillance systems focused on a broad range of snakebite-induced consequences including long-term pathological, psychological, and socioeconomic conditions.

**Conclusion:**

This case series describes pathological complications following cobra bites that require further research to determine mechanistic and epidemiological insights in the most affected regions by snakebites, specifically in India.

## Introduction

Snakebite envenoming causes a broad range of acute complications that need prompt medical attention to save lives and prevent disabilities [1]. Although research is limited, snakebite envenoming-induced long-term health, psychological and socioeconomic ramifications affect numerous patients [2–4]. Hence, scientific research on snakebite envenoming-induced long-term complications is critical for designing action plans to mitigate its burden. Here, we report four patients who developed squamous cell carcinoma several years after being bitten by cobras. Squamous cell carcinoma is a malignant tumor of epithelial cell origin with different etiology including chronic wounds [5]. Two cases of snakebite envenoming-associated carcinoma have been reported suggesting a potential link between chronic ulcerating wounds induced by venoms and the development of squamous cell carcinoma [6,7]. We present four patients who developed this complication, together with their investigations and treatment for squamous cell carcinoma.

## Case series

**Figure 1.**
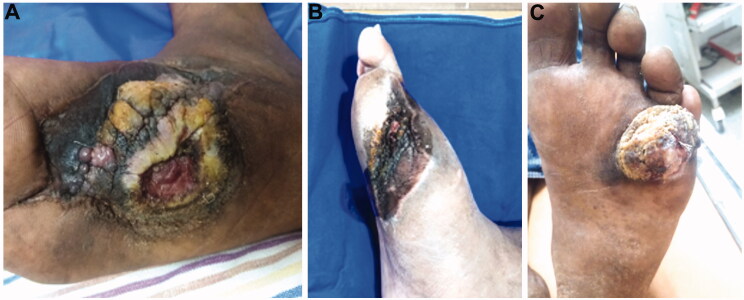
(A) Patient 1 displays a non-healing, fungating, ulcero-proliferative growth on the medial aspect of the right planter surface. (B) Patient 2 presents with a non-healing ulcer on the lateral aspect of the left foot. (C) Patient 4 has a fungating lesion on the sole of his left foot between the fourth and fifth toes.

A 58-year-old male was bitten by a suspected cobra 15 years previously on his right foot and received appropriate treatment in a local hospital. He developed bullae and extensive skin necrosis around the bite site for which multiple rounds of wound debridement were performed during his hospital stay (25 days). It took approximately 18 months for complete wound healing although a minimal area that was not covered by healthy skin desquamated frequently. Fourteen years after the bite, he again developed a minor ulcer at the same spot with a foul-smelling greenish-yellow discharge with skin sloughing. Examination in our hospital revealed a large ulcer on the right foot with a rolled edge and bulky soft tissue exophytic, red-grey mass (18 cm by 8 cm), which was fungating, with ulcero-proliferative growth with everted edges that was fixed to the underlying tissues along with an ulcer (Figure 1A).

A 42-year-old male was bitten by a cobra (the specimen was identified by a trained herpetologist) in his left foot 10 years previously and was treated in a local hospital where he developed multiple blisters and skin necrosis at the bite site. Therefore, radical excision of necrotic skin and regular dressing were performed, resulting in healing of most of the wounds although a small portion did not heal completely. Upon presentation in our hospital, examination revealed an irregularly shaped deep ulcer on the left foot measuring 14 cm by 6 cm. The wound exhibited a cauliflower-like pattern, and a thick layer foul-smelling necrotic material was observed (Figure 1B). The edge of the ulcer was hard and everted, approximately 1 cm higher than the surrounding skin.

A 52-year-old male was bitten by a suspected cobra 15 years previously in his right leg. He was treated in a local hospital where he had extensive swelling in the bitten leg, extending up to the mid-thigh and thus underwent a fasciotomy. However, the wounds at the bite site increased in size and worsened despite regular dressings and antibiotics. Therefore, surgical excision of the necrotic areas was performed, but the wounds did not heal and, 2 years later an ulcer developed with foul-smelling purulent discharge. Examination in our hospital revealed a firm ulcer (10 cm by 4 cm) over the dorsum of the right foot with swelling and serosanguineous discharge.

A 51-year-old male presented to our hospital with complaints of an enlarging, painful wart on the plantar surface of his left foot which was present for over 10 years. Fifteen years previously, he was bitten by a suspected cobra between his left fourth and little toe and developed an ulcer from a blister on the dorsum of his left foot. He underwent various treatments at local hospitals, but the ulcer continued to grow. Examination in our hospital revealed a soft tissue mass (4 cm by 3.5 cm by 1.5 cm) with a cauliflower-like fungating lesion on his left foot with a foul odor, and serosanguinous discharge (Figure 1C).

All patients failed to respond to antibiotic therapy and repeated dressings. Upon presentation (between March 2021 and December 2023), they were conscious, rationale and hemodynamically stable. Their routine investigations were normal. There was no bone tissue damage in any patients, but they displayed enlarged inguinal lymph nodes. All patients underwent a wedge biopsy of their wounds, and the histological analysis identified the presence of squamous cells infiltrating the dermis, confirming well-differentiated squamous cell carcinoma. A positron emission tomography scan confirmed the absence of metastasis to the lymph nodes. Surgical excision of tumor was performed in all patients, which included a 3 cm to 4 cm margin around the lesion that extended to the deep fascia. To assist wound healing three patients underwent a week of closed negative pressure suction therapy before their skin grafts, and one patient underwent a skin graft on the day of the excision. All grafts were well incorporated, and no further ulcers developed in the patients for up to 6 months of follow-up.

## Discussion

Cobra venoms mainly induce neurological manifestations, such as drowsiness, dyspnea, ptosis, paralysis and local tissue destruction [8] as was present in all four patients we report. However, the wounds did not heal completely following their initial treatments, and they continued to cause long-term complications. Although squamous cell carcinoma has several causes [5], we were only able to find two previous cases associated with envenomings from different snakes [6,7]. In both cases, squamous cell carcinoma developed several years later. The mechanisms behind the development of squamous cell carcinoma following envenoming remains poorly understood, although several studies have identified long-lasting ulcers, scars, and wounds as important risk factors for squamous cell carcinoma in non-envenomed patients [5]. While the cytotoxins present in cobra venoms induce tissue damage [9,10], in most cases, this responds to appropriate treatment. However, rarely, this can lead to long-term complications such as squamous cell carcinoma.

## Conclusion

This report demonstrates that chronic snakebite envenoming-related wounds may result in squamous cell carcinoma and emphasises the importance of long-term monitoring of such patients.
